# Photo images, 3D/CT data and mtDNA of the freshwater mussels (Bivalvia: Unionidae) in the Kyushu and Ryukyu Islands, Japan, with SEM/EDS analysis of the shell

**DOI:** 10.3897/BDJ.7.e32114

**Published:** 2019-01-28

**Authors:** Yuichi Kano, Yoshihisa Kurita, Kazuki Kanno, Kengo Saito, Hironori Hayashi, Norio Onikura, Takeshi Yamasaki

**Affiliations:** 1 Kyushu University, Fukuoka, Japan Kyushu University Fukuoka Japan; 2 Yamashina Institute for Ornithology, Konoyama, Japan Yamashina Institute for Ornithology Konoyama Japan

**Keywords:** 3D model, anatomy, CT scan, digital archiving, elemental composition, energy dispersive X-ray spectrometry (EDS), freshwater mussels morphology, open science, scanning electron microscope (SEM), shell exoskeleton

## Abstract

**Background:**

Freshwater mussels (Bivalvia: Unionidae), which are keystone species of freshwater ecosystems, are in global decline. In addition to ecological/genetic studies, morphological examinations are needed to help provide information for the development of additional freshwater mussel studies and eventually conservation efforts for freshwater ecosystems.

The microscopic structure, which can be obtained by scanning electron microscopy (SEM) and elemental composition, which can be obtained with energy dispersive X-ray spectrometry (EDS), of mollusc shells are of interest to malacologists. However, information about freshwater mussels is still limited.

Kyushu Island is the southernmost island of the four major islands of Japan. Kyushu Island is a hotspot of bitterling fishes in Japan, which simultaneously means that the island is a hotspot of freshwater mussels. The Ryukyu Islands stretch southwest from Kyushu Island to Taiwan; a freshwater mussel of unknown origin was reported from the Ryukyu Islands.

Digital archiving for biology and ecology is a continuing challenge for open science. This data paper describes online published photo images, 3D/CT and mtDNA data and SEM/EDS analyses of the shell of freshwater mussels that inhabit the Kyushu and Ryukyu Islands, Japan. Our data will provide basic information regarding freshwater biology and be of public interest as open science.

**New information:**

Photo images, 3D/CT data, mtDNA data, SEM images and EDS elemental analysis of freshwater mussels that inhabit the Kyushu and Ryukyu Islands (61 individuals, nine species/subspecies) were published online in a local database (http://ffish.asia/Unionidae3D), GBIF (http://ipt.pensoft.net/resource?r=unionidae3d) and DDBJ/EMBL/Genbank (LC431810–LC431840).

## Introduction

Freshwater mussels (Bivalvia: Unionidae) are one of the most endangered freshwater animals in Japan and around the world. In total, 21 unionid species/subspecies have been reported from Japan, including introduced *Hyriopsis cumingii* from China (Masuda and Uchiyama 2004, Imai 2008, Kondo 2008, Kitano and Imai 2012), of which 13 species/subspecies are listed as endangered (NT, VU or CR+EN) by the Red Data Book of the Ministry of the Environment, Japan (2018). Freshwater mussels have an ecological relationship with bitterling fishes, which are also generally threatened. Bitterling fishes lay their eggs in the gills of mussels; the life history/cycle of bitterling fishes is not completed without mussels (e.g. Kitamura et al. 2012). On the other hand, mussel larvae (glochidia) parasitise sympatric fishes, including bitterling fishes (Kondo 2008). Freshwater mussels also provide crucial ecosystem services by contributing to water clarity (e.g. Chowdhury et al. 2016). Thus, freshwater mussels are considered keystone species and more studies are needed to help provide information for conservation plans for freshwater ecosystems.

Kyushu Island is a hotspot for bitterling fishes in Japan (Onikura et al. 2011), which also means that the island is home to populations of freshwater mussels. The Ryukyu Islands stretch southwest from Kyushu Island to Taiwan; a freshwater mussel, *Cristaria tenuis*, was once reported from Ishigaki Island, one of the major Ryukyu Islands (Imai 2008). *Cristaria tenuis* was also found in Fukuoka Prefecture, which is in the northern part of Kyushu Island (Kitano and Imai 2012). Further studies are needed to determine whether *C. tenuis* is native to Ishigaki Island or Fukuoka Prefecture (Imai 2008).

Shell exoskeleton morphology is a crucial characteristic in mollusc studies related to hydrodynamics (e.g. Eagar 1978, Aldridge 1999, Denny and Blanchette 2000, Kondo 2008), whereas evaluation of the soft body has usually been overlooked. In addition to shell morphology, microscopic structure and chemical/elemental composition of the shell, which can be obtained by scanning electron microscopy (SEM) and energy dispersive X-ray spectrometry (EDS), have been of interest to malacologists (Matsuda and Hirano 1980, Carter 1990, Ubukata 1994, Fuchigami and Sasaki 2005, de Paula and Silveira 2006, Checa et al. 2016).

Digital archiving is one of the progressive challenges for the open science of biology and biodiversity studies (e.g. Berquist et al. 2012, Miyazaki et al. 2014, Kano et al. 2016, Kano et al. 2018). This paper describes photo images published online, 3D/CT data (to evaluate both the shell exoskeleton and soft body) and mtDNA data of freshwater mussels that inhabit the Kyushu and Ryukyu Islands of Japan (61 individuals of nine species/subspecies) with SEM images and EDS analysis of the shell. We expect our study will contribute valuable information to general malacology, freshwater ecosystem conservation and public interest in biodiversity conservation.

## Sampling methods

### Study extent

**Figure 1.**
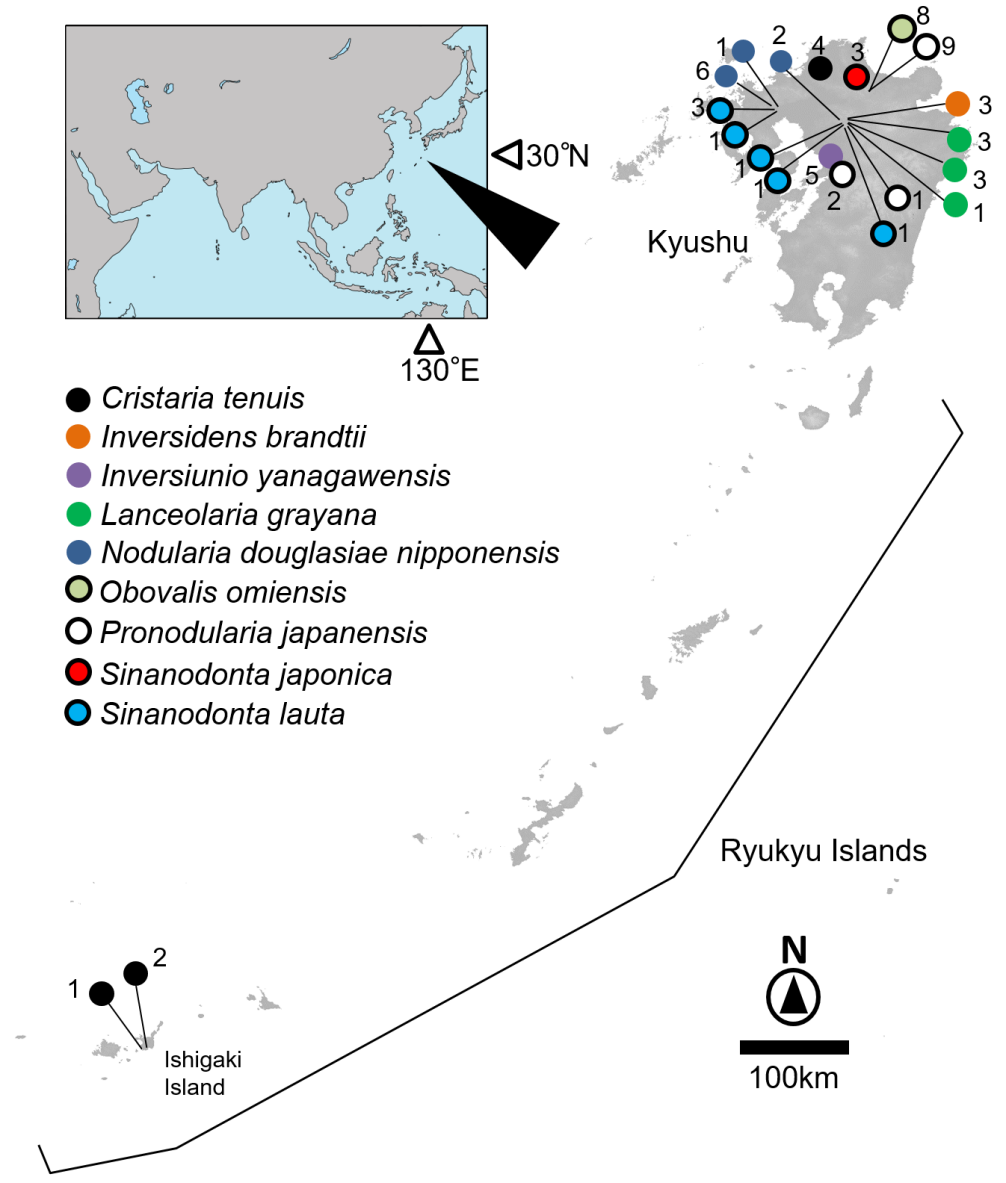
Sampling sites of the nine freshwater mussel species in the Kyushu and Ryukyu Islands. The number attached to each circle indicates the number of individuals.

Freshwater mussels were collected in the wild of Kyushu and Ryukyu Islands, Japan (Fig. 1).

### Sampling description

The living specimens (61 individuals) were captured by hand from the Tsuri, Tenkai, Yamakuni, Matsuura, Katsura, Chikugo, Kikuchi and Nagura River systems.

### Quality control

Identification followed Masuda and Uchiyama (2004), Kondo (2008) and Sano et al. (2017).

### Step description

**Figure 2.**
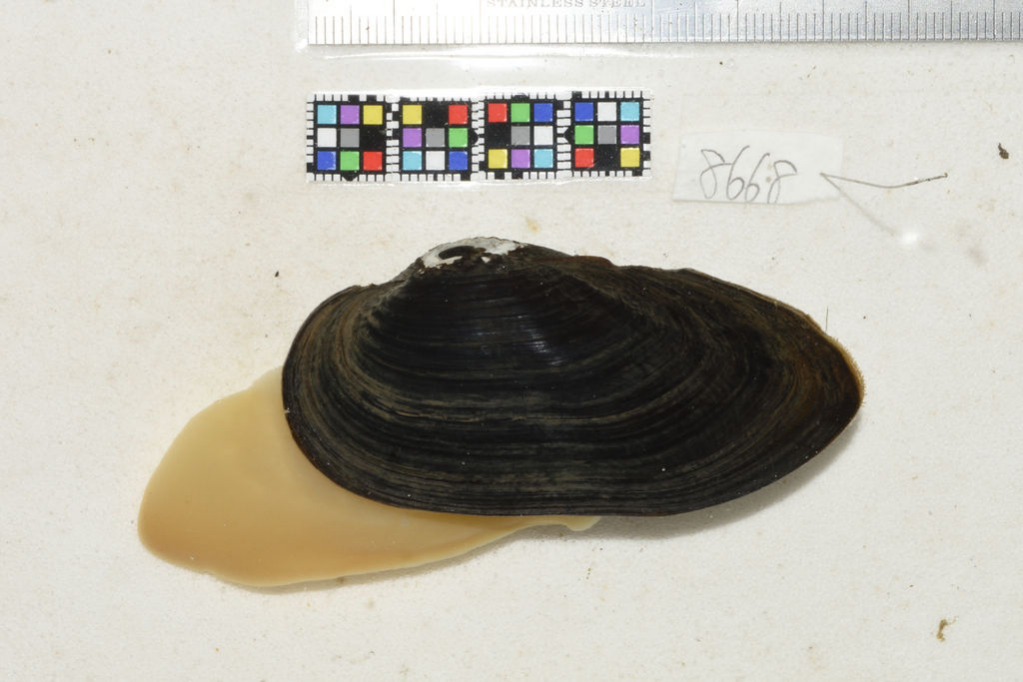
A live *Nodularia douglasiae nipponensis* (QUYK-08668d)

Individual photo images were taken in the field (Fig. 2) (Kano and Nakajima 2014). The specimens were fixed in 10% formalin followed by preservation in 70% ethanol. A small segment of the soft body was cut off and separately preserved in 99% ethanol for mtDNA analysis.

All specimens were CT scanned (Aloka Latheta LCT-200, Hitachi Ltd., Japan) and 3D surface models (Fig. 3; CT value: −450 to 600) were extracted from the CT data (Figs 4, 5).

**Figure 6.**
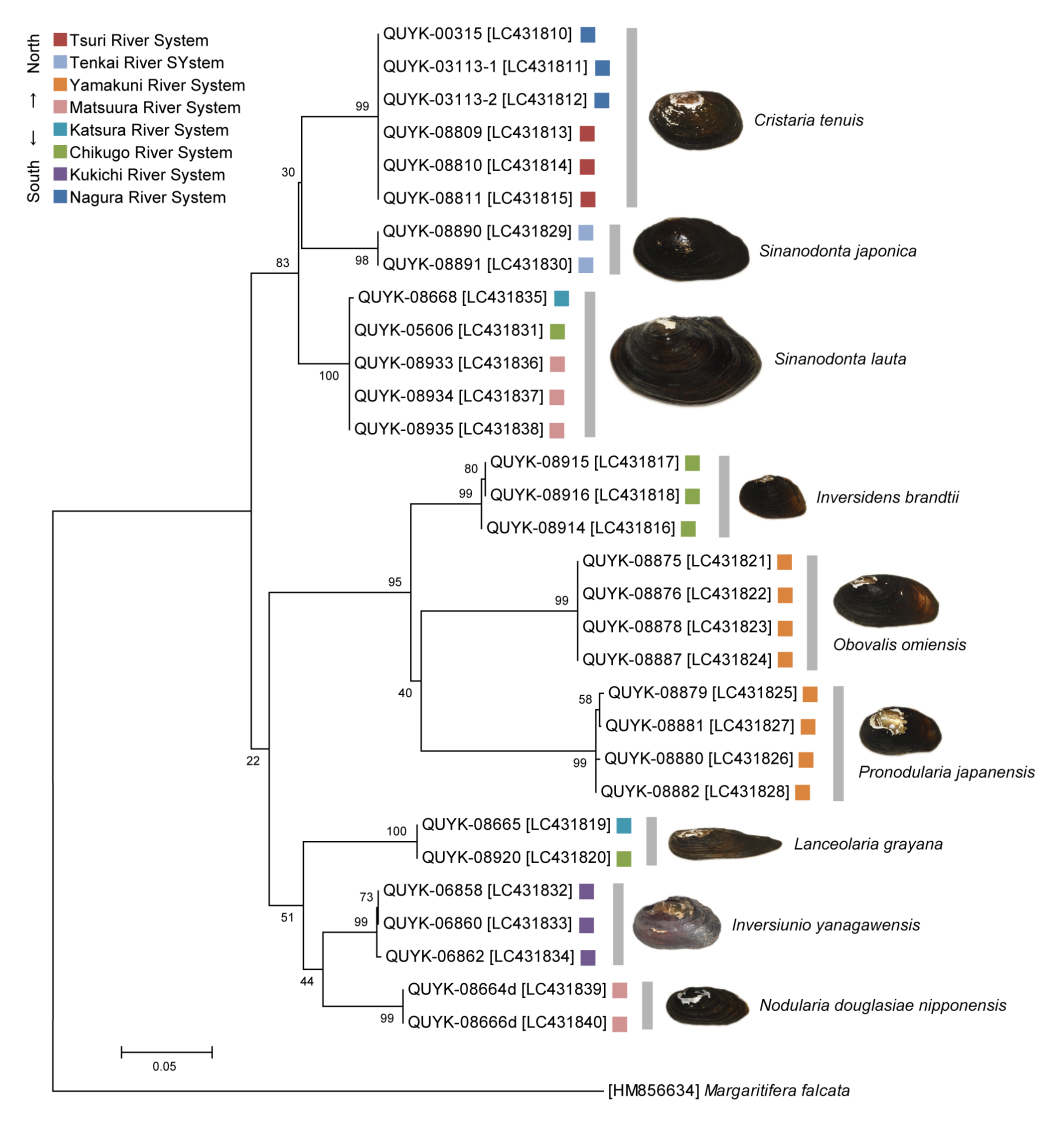
A phylogenetic tree of nine Unionidae species/subspecies found in the Kyushu and Ryukyu Islands (mtDNA 16S-rRNA). The tree was reconstructed using the maximum likelihood method (model: GTR+G) in MEGA 7 (Kumar et al. 2016). Bootstrap values were obtained with 500 pseudoreplicates. *Margaritifera falcata* was used as an outgroup. The DDBJ accession numbers are shown in brackets.

mtDNA analysis of 16S-rRNA was conducted for 31 individuals (Fig. 6). For PCR amplification, we used the primer pair Unio16SFwd (forward: 5′-TGCCTGTTTACCAAAAACATCG-3′) and Unio16SRev (reverse: 5′-CTTGGGGTCCTTTCGTACA-3′). PCR amplification was performed in 10-µl reaction mixtures that contained 5 µl KAPA 2G™ Robust HotStart ReadyMix (Kapa Biosystems, USA), 1 µM of each primer, 1 µl DNA template and 2 µl sterile deionised water. The reaction mixtures were preheated at 95°C for 3 min, followed by 30 amplification cycles (95°C for 15 s, 50°C for 15 s and 72°C for 40 s), with a final 5-min extension at 72°C. Direct sequencing of the PCR products was conducted externally (FASMAC, Japan). The nucleotide sequences were deposited in DDBJ/EMBL/GenBank (accession numbers: LC431810–LC431840).

The SEM/EDS analysis was conducted for 29 individuals. A shell fragment was cut off from each specimen (from the posterior part of the shell) and the inner side of the shell was analysed by SEM (JCM-6000, JEOL Ltd., Japan) to observe the microscopic images of the pearled surface (Fig. 7). Furthermore, EDS analysis (JED-2300, JEOL Ltd.) was conducted to determine the elemental composition of the shell fragment by targeting B, C, N, O, F, Na, Mg, Al, Si, P (Fig. 8a), S, Cl, K and Ca (Fig. 8b) (Suppl. material 1).

## Geographic coverage

### Description

Freshwater habitats of Kyushu and Ryukyu Islands, Japan.

### Coordinates

24.4 and 33.8 Latitude; 124.2 and 131.2 Longitude.

## Taxonomic coverage

### Description

All freshwater mussels (family Unionidae), distributed in the Kyushu and Ryukyu Islands, were studied except *Anemina arcaeformis*, *Hyriopsis schlegelii* and *Sinanodonta* sp. A shell exoskeleton, that was photographically identified as *A. arcaeformis*, was reported from Miyazaki Prefecture, Kyushu Island (Toyama and Nishi 2016), although all other details remain unclear. *Hyriopsis schlegelii* individuals were artificially introduced to Isahaya Bay for water purification (Ishizaki et al. 2007), even though *H. schlegelii* is non-native to Kyushu Island. *Sinanodonta* sp. (or spp.) populations were informally reported from the Ryukyu Islands, but some of the populations were likely introduced from Taiwan (Shokita 1984, Kurozumi 2003, Imai 2008).

### Taxa included

**Table:** 

Rank	Scientific Name	Common Name
kingdom	Animalia	Animals
phylum	Mollusca	Molluscs
class	Bivalvia	Bivalves
order	Unionoida	Freshwater mussels and pearl mussels
family	Unionidae	Freshwater mussels
species	*Cristaria tenuis*	"Dobu-gai-modoki"
species	*Inversidens brandtii*	"Oba-eboshi-gai"
species	*Inversiunio yanagawensis*	"Nise-matsukasa-gai"
species	*Lanceolaria grayana*	"Tongari-sasanoha-gai"
subspecies	*Nodularia douglasiae nipponensis*	"Ishi-gai"
species	*Obovalis omiensis*	"Kataha-gai"
species	*Pronodularia japanensis*	"Matsukasa-gai"
species	*Sinanodonta japonica*	"Ta-gai"
species	*Sinanodonta lauta*	"Numa-gai"

## Temporal coverage

### Notes

From 2013-12-21 to 2018-06-29.

## Usage rights

### Use license

Other

### IP rights notes

CC BY-NC 4.0

## Data resources

### Data package title

Photo images, 3D/CT data and mtDNA of the freshwater mussels (Bivalvia: Unionidae) in the Kyushu and Ryukyu Islands, Japan, with SEM/EDS analysis of the shell.

### Number of data sets

2

### Data set 1.

#### Data set name

Unionidae3D

#### Data format

html; jpg; Wavefront object format (.obj); CT dicom file (.dcm); text for mtDNA sequence.

#### Number of columns

11

#### Character set

UTF8

#### Download URL

http://ffish.asia/Unionidae3D

#### Description

Photo images, surface 3D models and CT scanned data are available for 61 individuals. To render the CT dicom files as a visual 3D volume, several free software are available. mtDNA sequences (16S-rRNA) are also available for 31 individuals. SEM images and EDS analysis are available for 29 individuals. Below, the main 11 columns are listed;

**Data set 1.** 

Column label	Column description
Specimen/Data ID	ID for the specimen
Images	Images for specimen and associated files
Species	Species information
Taxon	Order, family and genus
DNA	DNA sequence data if available
N	Number of individuals
Country	Country where the sample was obtained
Sampling location	Description of the locality where the specimen was obtained with a map (the resolution of locality data is shown roughly to prevent poaching)
Location	Locality information
Habitat	Habitat information
Comment	Other information

### Data set 2.

#### Data set name

Photo images, 3D/CT data and mtDNA of the freshwater mussels (Bivalvia: Unionidae) in the Kyushu and Ryukyu Islands, Japan, with SEM/EDS analysis of the shell

#### Data format

Darwin Core Archive

#### Number of columns

42

#### Download URL

http://ipt.pensoft.net/resource?r=unionidae3d

#### Description

GBIF registered occurrence and multimedia data for the specimens. Below, the 42 columns are listed;

**Data set 2.** 

Column label	Column description
occurrenceID (occurrence)	Occurrence ID
basisOfRecord (occurrence)	The specific nature of the data record
eventDate (occurrence)	The date-time or interval during which the specimen collected
year (occurrence)	The four-digit year in which the Event occurred, according to the Common Era Calendar
month (occurrence)	The ordinal month in which the Event occurred
day (occurrence)	The integer day of the month on which the Event occurred
eventRemarks (occurrence)	Comments or notes about the Event
scientificName (occurrence)	Scientific name (or tentative name) of the specimen
kingdom (occurrence)	The full scientific name of the kingdom in which the taxon is classified
phylum (occurrence)	The full scientific name of the phylum or division in which the taxon is classified
class (occurrence)	The full scientific name of the class in which the taxon is classified
order (occurrence)	The full scientific name of the order in which the taxon is classified
family (occurrence)	The full scientific name of the family in which the taxon is classified
genus (occurrence)	The full scientific name of the genus in which the taxon is classified
specificEpithet (occurrence)	The name of the first or species epithet of the scientificName
infraspecificEpithet (occurrence)	The name of the lowest or terminal infraspecific epithet of the scientificName, excluding any rank designation
taxonRank (occurrence)	The taxonomic rank of the most specific name in the scientificName. Recommended best practice is to use a controlled vocabulary
nomenclaturalCode (occurrence)	The nomenclatural code (or codes in the case of an ambiregnal name) under which the scientificName is constructed
decimalLatitude (occurrence)	Value of decimal latitude (0.1 degree level: the resolution of locality data is shown roughly to prevent poaching)
decimalLongitude (occurrence)	Value of decimal longitude (0.1 degree level: the resolution of locality data is shown roughly to prevent poaching)
geodeticDatum (occurrence)	The ellipsoid, geodetic datum or spatial reference system (SRS) upon which the geographic coordinates given in decimalLatitude and decimalLongitude as based
coordinateUncertaintyInMeters (occurrence)	The horizontal distance (in metres) from the given decimalLatitude and decimalLongitude describing the smallest circle containing the whole of the Location
verbatimCoordinateSystem (occurrence)	The spatial coordinate system for the verbatimLatitude and verbatimLongitude or the verbatimCoordinates of the Location
islandGroup (occurrence)	The name of the island group in which the Location occurs
island (occurrence)	The name of the island on or near which the Location occurs
country (occurrence)	The name of the country or major administrative unit in which the Location occurs
countryCode (occurrence)	The standard code for the country in which the Location occurs
stateProvince (occurrence)	The name of the next smaller administrative region than country (state, province, canton, department, region etc.) in which the Location occurs
locality (occurrence)	The specific description of the place
individualCount (occurrence)	The number of individuals represented present at the time of the Occurrence
establishmentMeans (occurrence)	The process by which the biological individual(s) represented in the Occurrence became established at the location
preparations (occurrence)	Preservation methods for a specimen
occurrenceID (multimedia)	Occurrence ID
references (multimedia)	An html webpage that shows the image or its metadata
title (multimedia)	The media items title
type (multimedia)	The kind of media object
format (multimedia)	The format the image is exposed in
description (multimedia)	A textual description of the content of the media item
created (multimedia)	The date and time this media item was taken
creator (multimedia)	The person who took the image, recorded the video or sound
audience (multimedia)	A class or description for whom the image is intended or useful
rightsHolder (multimedia)	A person or organisation owning or managing rights over the media item

## Additional information

### Note on the origin of *Cristaria tenuis*

We cannot conclude whether *Cristaria tenuis* is native or introduced based on our results (Fig. 6). However, it is notable that the sequences were all the same amongst the six samples from the two distant locales; *C. tenuis* might have been introduced in either Ishigaki Island or Fukuoka Prefecture.

### “Metal artefacts” in CT data of thick-shell individuals

Several individuals, especially *Inversiunio yanagawensis* individuals, had significantly thicker shells; streak-like artefacts were generated at the surface of the shell as noise and were considered “metal artefacts” (e.g. Fig. 3a).

## Supplementary Material

Supplementary material 1 Elemental composition of the shell by EDS Data type: csv
File: oo_242175.csv Yuichi Kano

## Figures and Tables

**Figure 3a.**
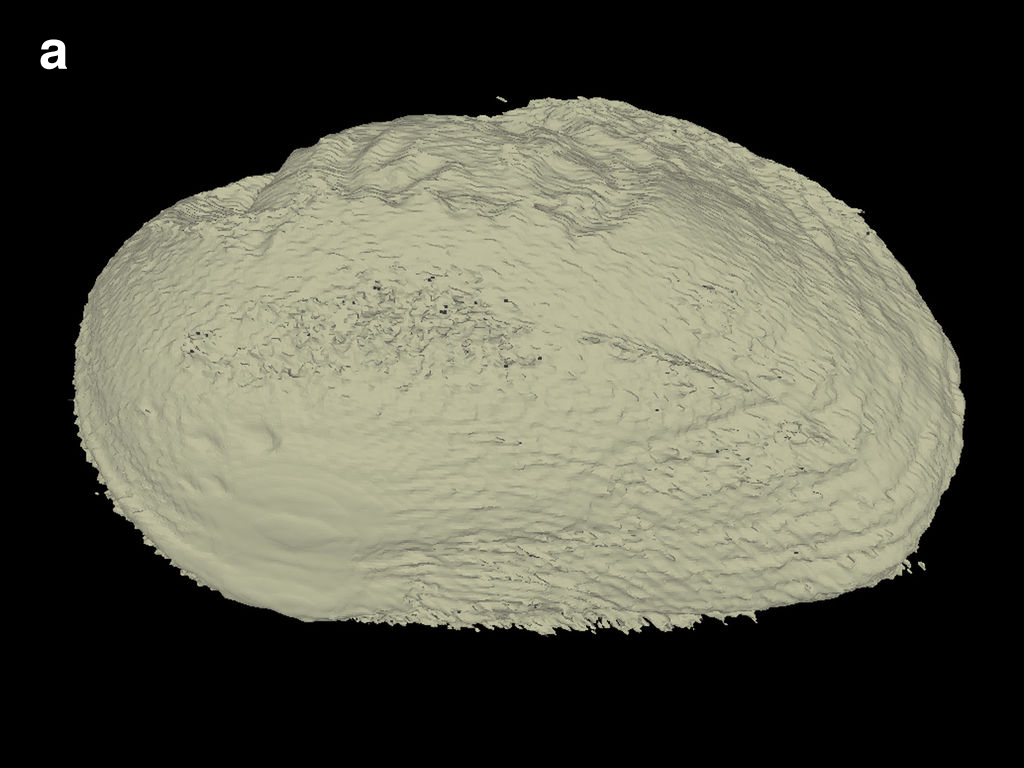
*Inversiunio yanagawensis* (QUYK-06858) with "metal artifacts" noise due to the thick/hard shell.

**Figure 3b.**
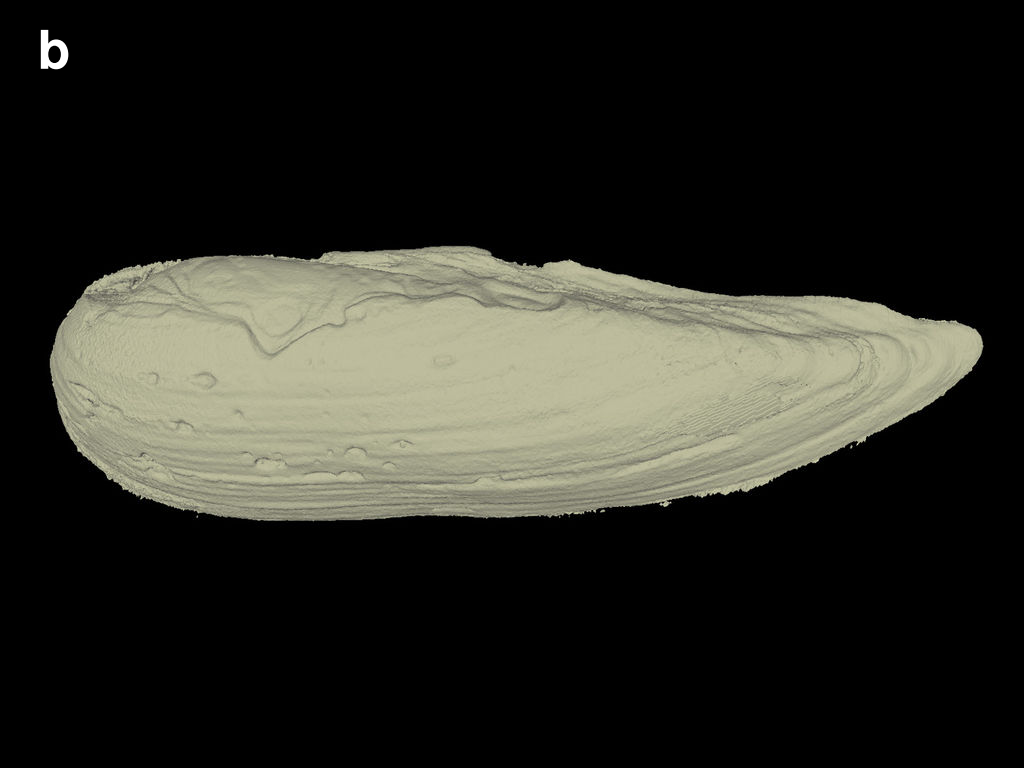
*Lanceolaria grayana* (QUYK-08773d).

**Figure 3c.**
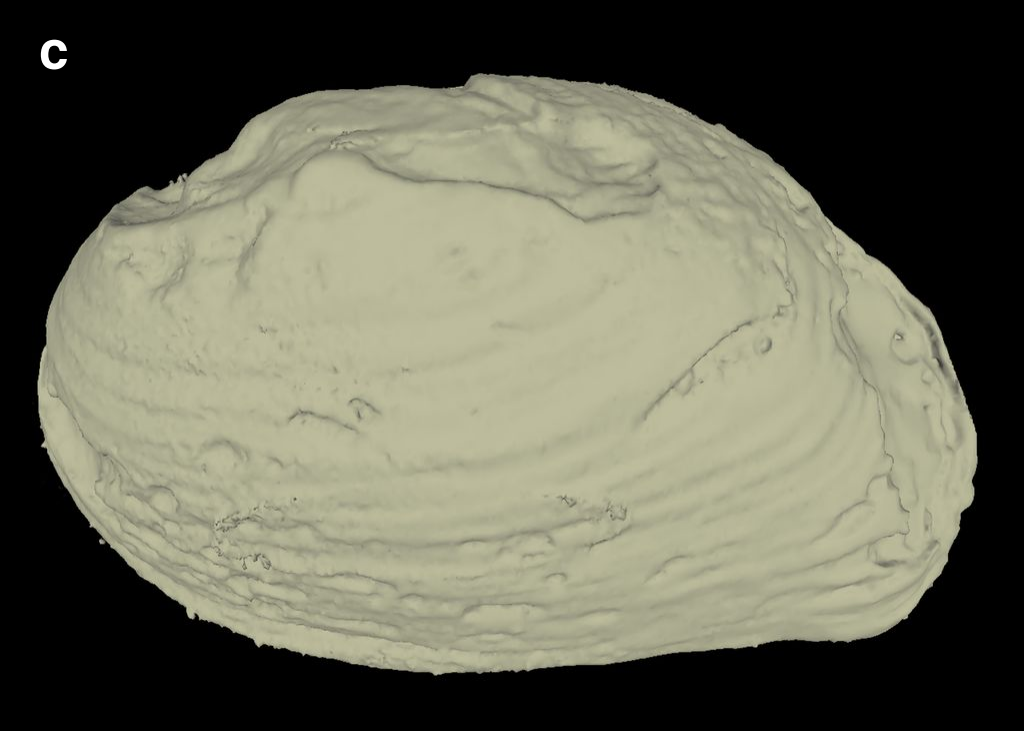
*Pronodularia japanensis* (QUYK-08881).

**Figure 3d.**
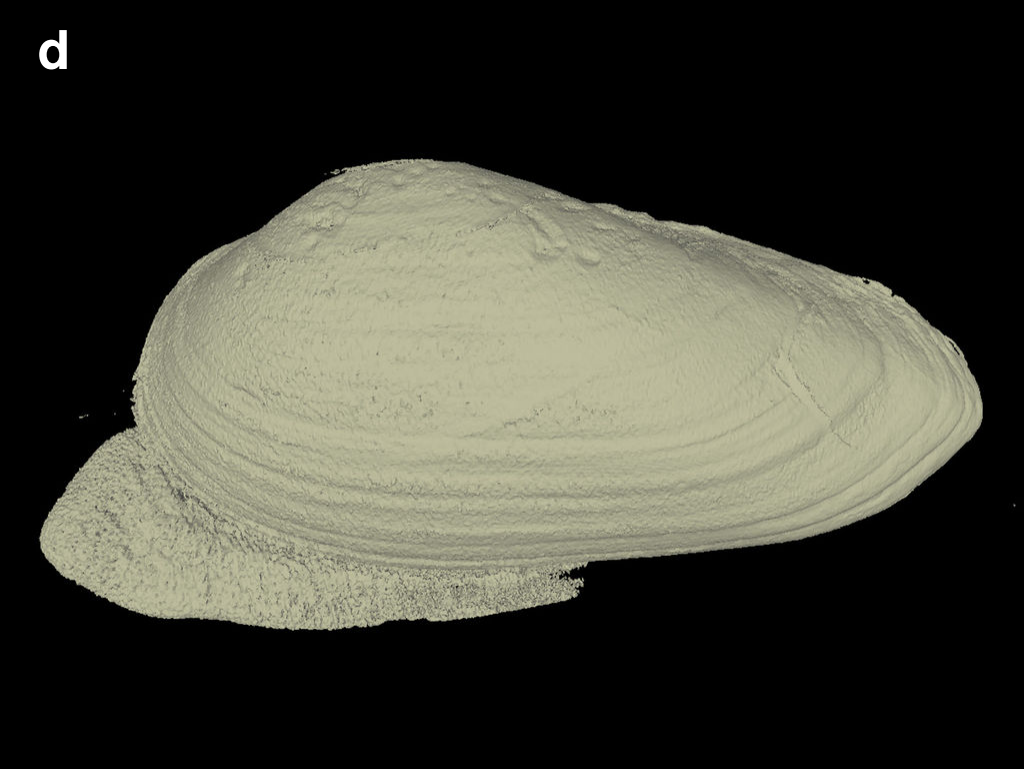
*Nodularia douglasiae nipponensis* (QUYK-08667d).

**Figure 4a.**
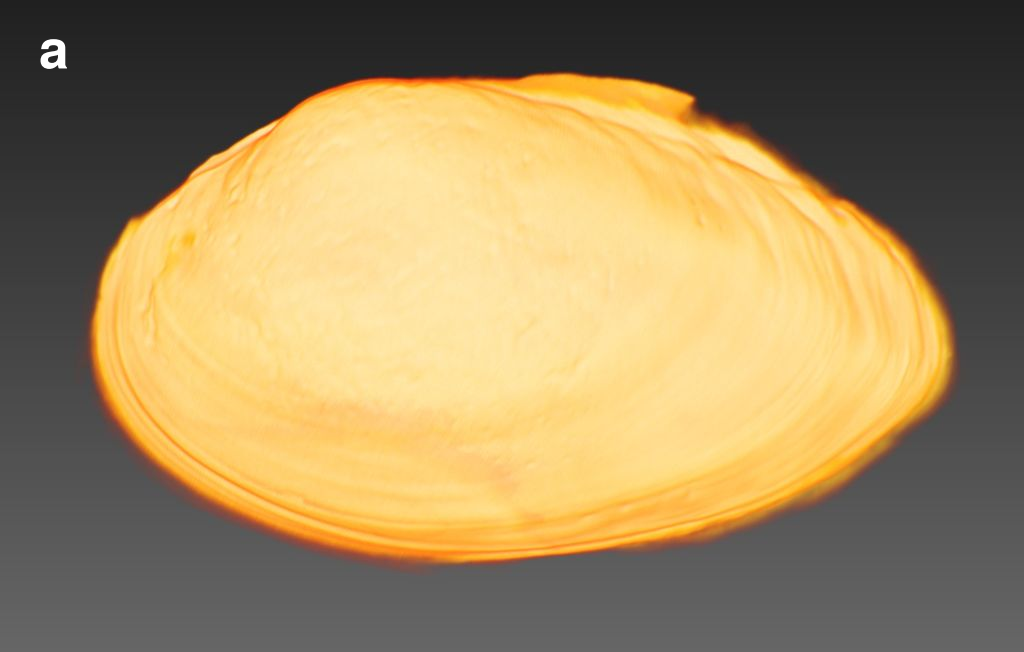
External shell exoskeleton.

**Figure 4b.**
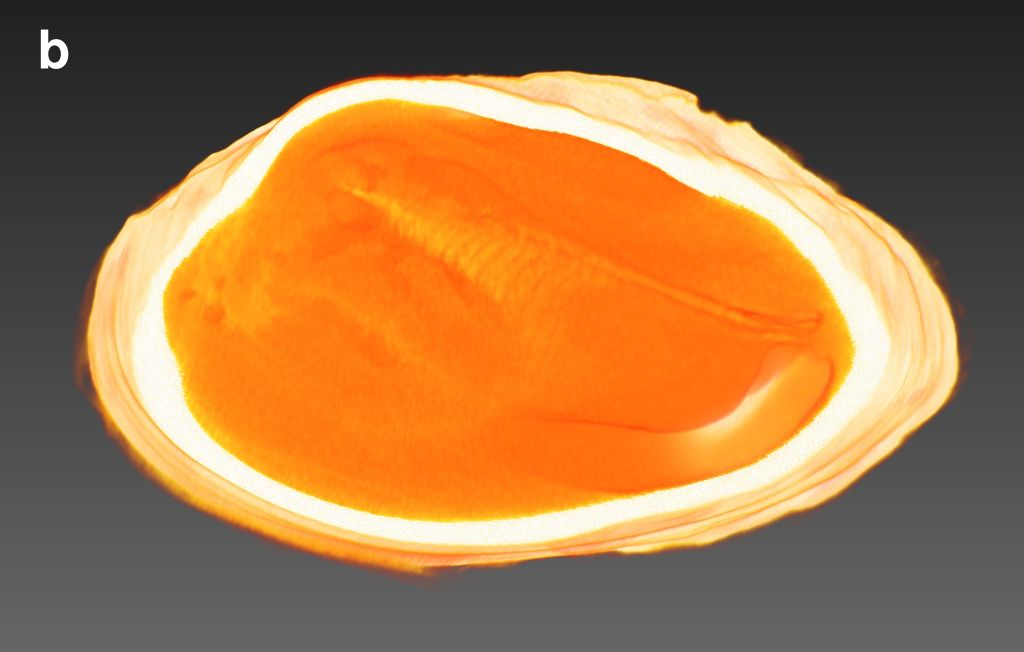
Digitally sliced shell that shows the internal soft body.

**Figure 5.** A CT-scanned image of *Obovalis omiensis* (QUYK-08887).

**Figure 7a.**
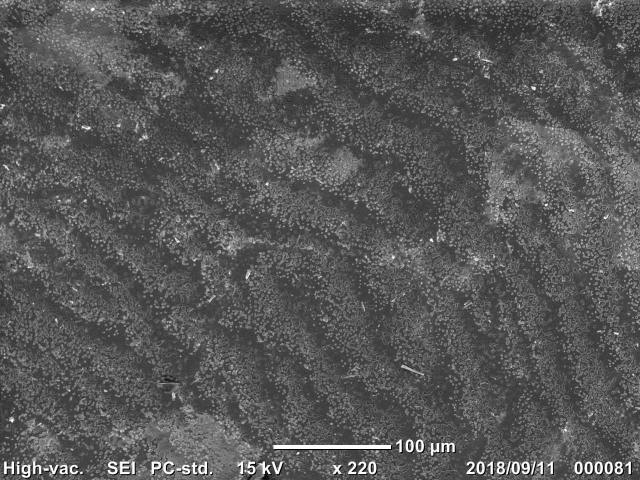
Multilevel wavelike structure of *Sinanodonta japonica* QUYK-08891 (Scale bar 100 μm).

**Figure 7b.**
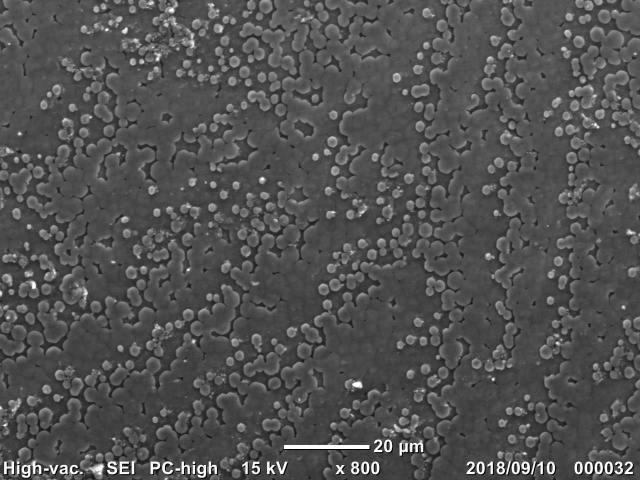
Multilevel wavelike structure of *Obovalis omiensis* QUYK-08872 (Scale bar 20 μm).

**Figure 7c.**
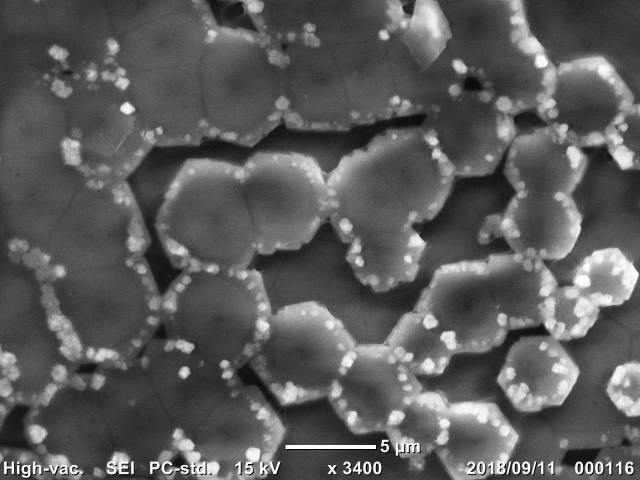
Hexagonal microstructure of *Inversidens brandtii* QUYK-08915 (Scale bar 5 μm).

**Figure 7d.**
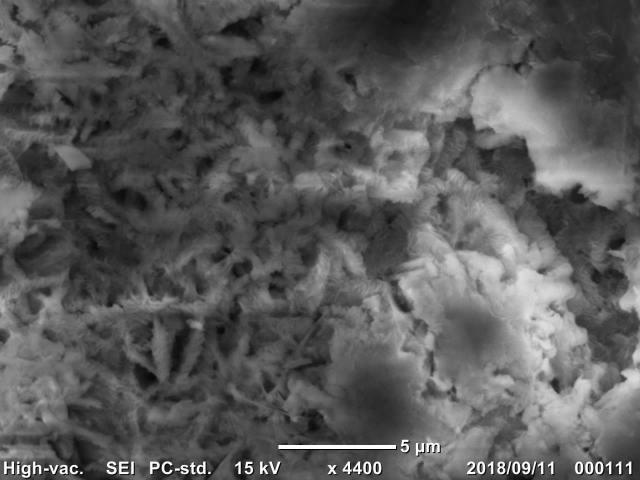
Columnar microstructure of *Pronodularia japanensis* QUYK-08880 (Scale bar 5 μm).

**Figure 8a.**
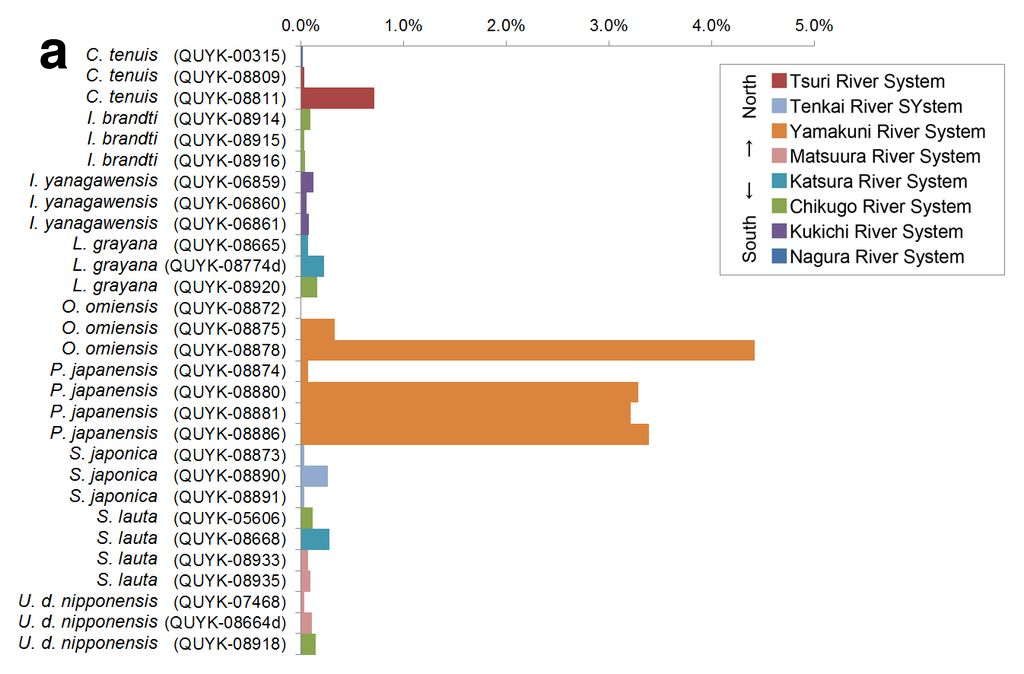
Phosphorous.

**Figure 8b.**
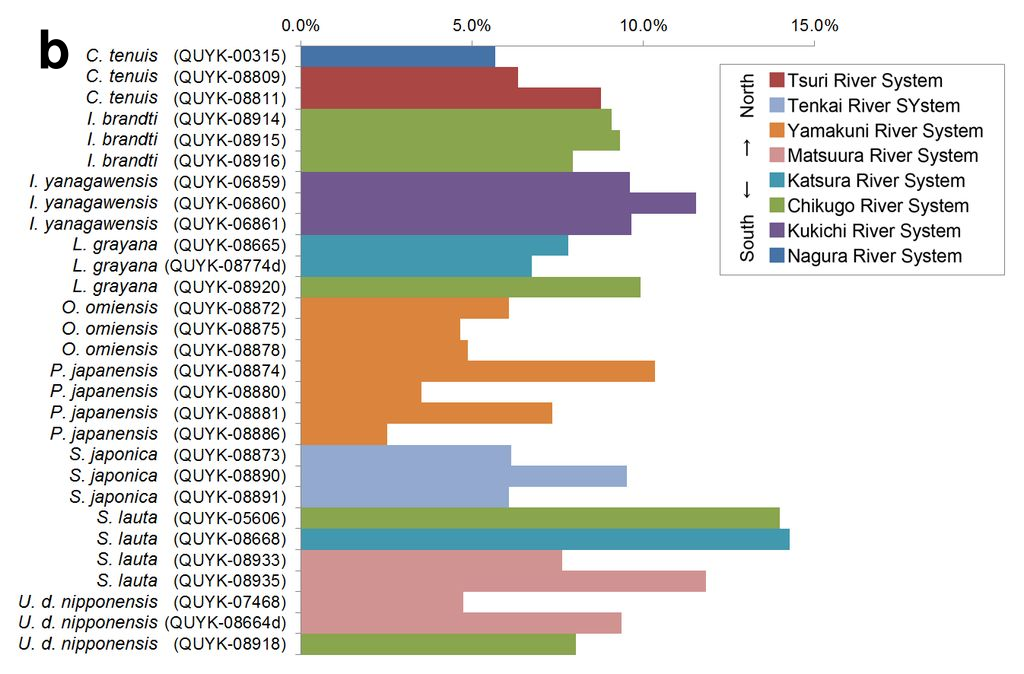
Calcium.
